# Synsor: a tool for alignment-free detection of engineered DNA sequences

**DOI:** 10.3389/fbioe.2024.1375626

**Published:** 2024-07-12

**Authors:** Aidan P. Tay, Kieran Didi, Anuradha Wickramarachchi, Denis C. Bauer, Laurence O. W. Wilson, Maciej Maselko

**Affiliations:** ^1^ Australian e-Health Research Centre, Commonwealth Scientific and Industrial Research Organisation (CSIRO), Sydney, NSW, Australia; ^2^ Applied Biosciences, Faculty of Science and Engineering, Macquarie University, Sydney, NSW, Australia; ^3^ Health and Biosecurity, Commonwealth Scientific and Industrial Research Organisation (CSIRO), Sydney, NSW, Australia

**Keywords:** alignment-free, engineered DNA, deep learning, biosecurity, biothreat detection

## Abstract

DNA sequences of nearly any desired composition, length, and function can be synthesized to alter the biology of an organism for purposes ranging from the bioproduction of therapeutic compounds to invasive pest control. Yet despite offering many great benefits, engineered DNA poses a risk due to their possible misuse or abuse by malicious actors, or their unintentional introduction into the environment. Monitoring the presence of engineered DNA in biological or environmental systems is therefore crucial for routine and timely detection of emerging biological threats, and for improving public acceptance of genetic technologies. To address this, we developed Synsor, a tool for identifying engineered DNA sequences in high-throughput sequencing data. Synsor leverages the k-mer signature differences between naturally occurring and engineered DNA sequences and uses an artificial neural network to classify whether a DNA sequence is natural or engineered. By querying suspected sequences against the model, Synsor can identify sequences that are likely to have been engineered. Using natural plasmid and engineered vector sequences, we showed that Synsor identifies engineered DNA with >99% accuracy. We demonstrate how Synsor can be used to detect potential genetically engineered organisms and locate where engineered DNA is being introduced into the environment by analysing genomic and metagenomic data from yeast and wastewater samples, respectively. Synsor is therefore a powerful tool that will streamline the process of identifying engineered DNA in poorly characterized biological or environmental systems, thereby allowing for enhanced monitoring of emerging biological threats.

## 1 Introduction

DNA sequences of nearly any desired composition, length, and function can be engineered (Hughes and Ellington, 2017). In this process, novel sequences are designed with computational tools and constructed with DNA synthesis and assembly techniques. In doing so, DNA sequences ranging from short oligonucleotides to whole genomes, can be engineered for use in different applications including disease treatment, drug production, bioremediation, and invasive pest control (Wang and Zhang, 2019). Yet despite offering many great benefits, engineered DNA poses a risk due to their possible misuse or abuse by malicious actors (i.e., bioterror), or their unintentional introduction into the environment (i.e., bioerror). Monitoring the presence of engineered DNA in biological or environmental systems is therefore crucial for detecting emerging biological threats (i.e., biothreats) arising from bioterror and bioerror (Wang and Zhang, 2019).

In many biosecurity contexts, identifying engineered DNA in biological or environmental systems remains challenging. This is because the identity of the engineered DNA is unknown, or the system is not well characterized (Ruttink et al., 2010; Fraiture et al., 2015). Instead, entire genomes or metagenomes worth of read data produced by high-throughput sequencing methods must be analyzed to identify engineered DNA sequences, which may involve specialized workflows or significant manual processing (Gargis et al., 2019; Buytaers et al., 2021; Collins et al., 2021). New methods that enable a more targeted approach to identifying engineered DNA in biological and environmental systems are therefore needed to streamline the process.

Alignment-free approaches can be used to distinguish between sequences originating from different species (Tay et al., 2021). These approaches typically involve characterizing sequences based on their oligonucleotide frequencies (referred to as k-mer signature) and evaluating the similarity between these k-mer signatures (Zielezinski et al., 2017). Closely related sequences will produce similar k-mer signatures, while distantly related sequences will have more distinct k-mer signatures (Karlin et al., 1997). Accordingly, engineered DNA sequences may contain different k-mers and therefore k-mer signatures that are sufficiently different to natural DNA sequences (Allen et al., 2008). At the same time, these k-mer signatures may serve as a useful representation for classifying DNA via pattern recognition algorithms such as neural networks. Querying such models could therefore serve as a strong prefilter, enabling the rapid identification of engineered DNA sequences in entire genomes or even a collection of metagenomes.

In this study, we developed Synsor, a tool for identifying engineered DNA sequences in high-throughput sequencing data. Synsor leverages k-mer signature differences between naturally occurring and engineered DNA sequences and uses an artificial neural network to classify whether a DNA sequence is natural or engineered. By querying suspected sequences against the model, Synsor can identify sequences that are likely to have been engineered. To demonstrate how Synsor can be used to identify engineered DNA in biological and environmental systems, we present case studies from yeast and wastewater samples.

## 2 Materials and methods

### 2.1 Dataset preparation

Natural plasmid and engineered vector sequences were obtained via the FTP server of NCBI. To account for differences in sequence coverage and provide a clear basis for comparison, training, and evaluation of predictive models, only sequences that were full-length were used in this study. Sequences were considered full-length if their FASTA header line contained either “complete sequence” or “complete genome.” To account for the unequal distribution of sequences between classes and within each sequence class (Supplementary Figure S1), full-length sequences that were longer than 20 kb were removed. Full-length sequences shorter than 2.5 kb were also removed to ensure that the k-mer signatures of sequences were stable. Together, this resulted in a total of 8,739 natural plasmid and 9,735 engineered vector sequences.

### 2.2 Sequence encoding

DNA sequences were encoded into fixed-length frequency vectors (referred to as k-mer signatures) using a custom script. This was done by identifying all possible subsequences of a given length (i.e., k-mer) and counting the frequency of each k-mer. k-mers containing ambiguous bases (i.e., N’s) were removed. The frequency of each k-mer and its reverse complement were then summed to reduce the size of the frequency vectors. Following this, we calculated the relative proportion of each k-mer, resulting in a relative frequency vector for each sequence. An overview of the workflow used to calculate k-mer signatures is shown in Supplementary Figure S2. The k-mer signatures of sequences were then used in subsequent analyses.

### 2.3 Identification of engineered DNA sequences using Synsor

Synsor (v1.00) was developed in Python and is used as a command-line tool. The source code is available under the GPL v3 license via the GitHub: https://github.com/aidantay/Synsor. A full description of Synsor is described in the Results section.

To identify engineered DNA sequences, Synsor uses an artificial neural network to classify whether a DNA sequence is natural or engineered (i.e., binary classification). An artificial neural network was used because they can capture complex non-linear relationships. Preliminary findings also showed that a multi-layered perceptron (i.e., neural network) performed well (Supplementary Figure S3) compared to other classifiers (i.e., uniform sampling, Logistic Regression, Gaussian Naïve Bayes, Random Forest, K-Nearest Neighbors). To train and evaluate the performance of the model, natural plasmid and engineered vector sequences from NCBI were first randomly partitioned into an 80% training set and 20% testing set following standard machine learning practice. Natural plasmid and engineered vector sequences were used because they are well-documented in public databases and known to be naturally occurring or artificial, respectively. The model was trained on the k-mer signatures of sequences in the training set, to predict whether a sequence belongs to the “engineered” class. After training on the full training set, we evaluated the performance of the model in predicting the “engineered” class using the k-mer signatures of sequences in the testing set.

The final model configuration and hyperparameters were obtained using 5-fold cross-validation, whereby 1-fold of the training set was used to validate the model. The model with the highest accuracy was used as the final model. It consists of an input layer, two fully connected hidden layers with 512 and 16 neurons respectively, and an output layer with 1 neuron. To prevent overfitting, dropout layers with 0.2 and 0.5 probabilities were inserted after the input layer and after each hidden layer, respectively. The rectified linear unit (ReLU) and sigmoid activation functions were used for the hidden and output layers, respectively. Loss was computed by binary cross-entropy and Adam was used as the optimizer.

### 2.4 Experimental genomic data

Genomic data for natural yeast (Giordano et al., 2017) and genetically engineered yeast (Collins et al., 2021) were obtained from the European Nucleotide Archive. Giordano et al. sequenced the genome of wild-type *Saccharomyces cerevisiae* strain S288C on an Illumina MiSeq sequencing platform (ENA project accession number PRJEB19900). Meanwhile, Collins et al. transformed different laboratory and nonconventional yeast strains with different engineered vector constructs, resulting in the construction of 15 engineered yeast strains. After growing transformed yeast cultures, Collins et al. sequenced the genome of each engineered yeast strain on an Illumina iSeq 100 sequencing platform (ENA project accession number PRJNA650312).

### 2.5 Simulated metagenomic data

To simulate the introduction of a genetically engineered organism to the environment, we constructed a synthetic metagenomic dataset. To do this, we combined metagenomic samples taken from wastewater treatment plants (Che et al., 2019) with genomic data for a genetically engineered bacterium (Ames et al., 2019). Che et al. sequenced the metagenomes of bacteria in influent samples taken at wastewater treatment plants in three different geographical locations around Hong Kong, namely, Shatin, Shek Wu Hui and Stanley (ENA run accession SRR8208343, SRR8208344 and SRR14455375). Meanwhile, Ames et al. sequenced the genome of *Escherichia coli* cells (strain BL21 DE3 pLysS) transformed with a pRSF expression vector containing the ParE toxin from *Mycobacterium tuberculosis* (ENA run accession number SRR9304539).

## 3 Results

### 3.1 Investigating differences between natural and engineered DNA

To investigate the differences between natural and engineered DNA, we first analysed 8,739 natural plasmid and 9,735 engineered vector sequences obtained from NCBI. Sequences were converted into k-mer signatures using different values of k (ranging from 3 to 9). These values for k were chosen to balance the trade-off between sequence specificity and computing requirements since longer k-mers can lead to prohibitively high computational resources. Finally, for each value of k, we performed Principal Component Analysis (PCA) on the k-mer signatures of natural plasmid and engineered vector sequences.

**FIGURE 1:**
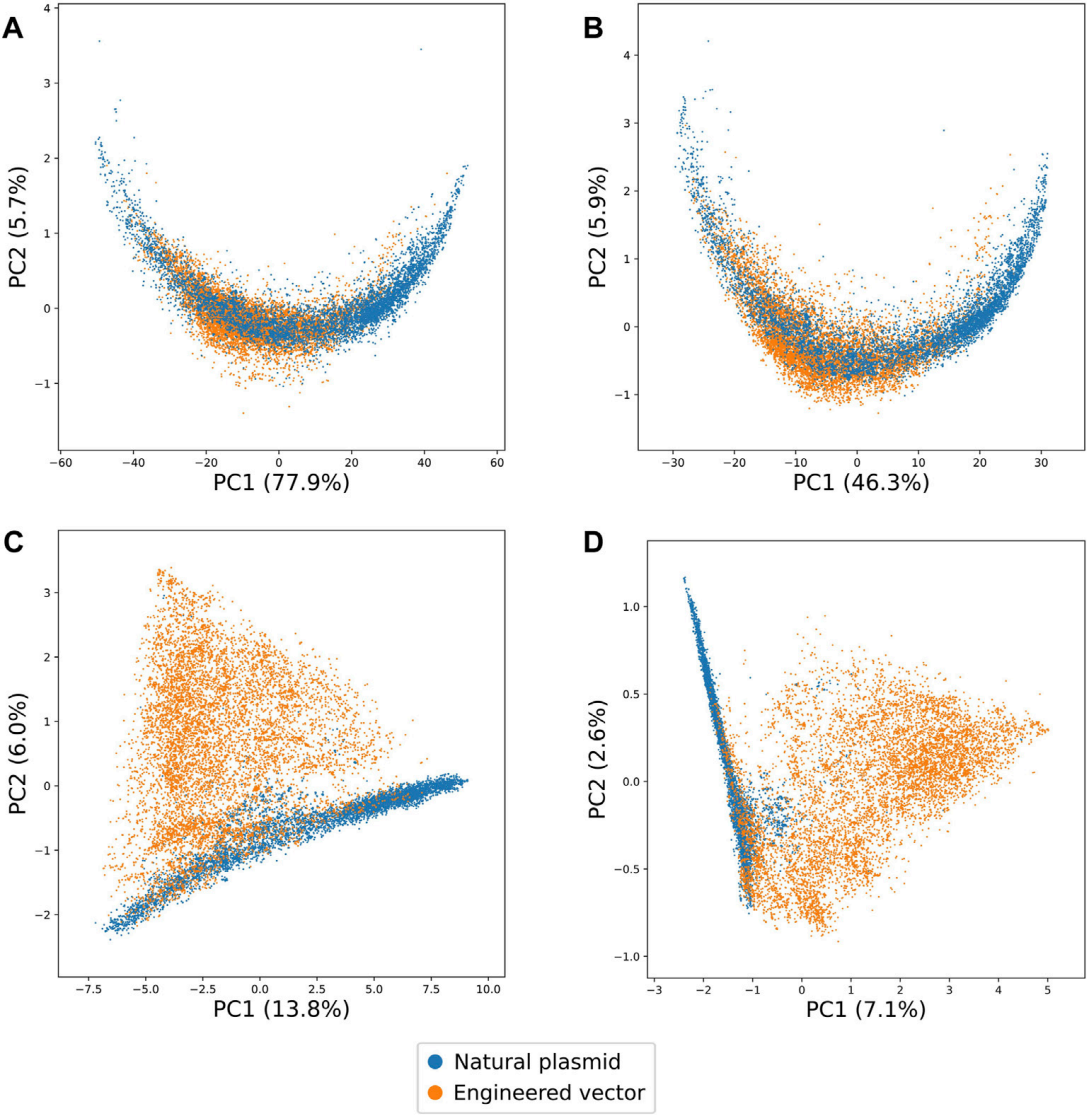
Two-dimensional representations learnt by PCA on the k-mer signatures of natural plasmid and engineered vector sequences for different values of k. The proportion of variances explained by the first two principal components are shown in brackets. **(A)** 3-mer, **(B)** 5-mer, **(C)** 7-mer and **(D)** 9-mer signatures of natural plasmid and engineered vector sequences are highlighted in blue and orange, respectively. Overall, increasing the value of k increased the separability of natural plasmid and engineered vector sequences.

Upon visualisation of the first two principal components for each value of k, we found that increasing the value of k increased the separability of natural plasmid and engineered vector sequences (Figure 1). Notably, with 3-mer and 5-mer signatures, we observed natural plasmid sequences overlapping with engineered vector sequences. By contrast, with 7-mer and 9-mer signatures, we observed less overlap between natural plasmid and engineered vector sequences. Furthermore, we noted that increasing the value of k increased the number of principal components required to represent at least 90% of the total variance (Supplementary Figure S4). The total number of principal components required to represent at least 90% of the total variance ranged between 5 (for 3-mer) and 3,341 (for 9-mer), whereby the total variance represented by the first two principal components ranged between 83.6% (for 3-mer) and 9.6% (for 9-mer). Thus, with sufficiently long k-mers (i.e., ≥ 7-mers), the above suggests that natural and engineered DNA are distinct sequence classes that have unique k-mer signatures. However, with shorter k-mers (i.e., ≤ 5-mers), the lack of distinct k-mer signatures for natural and engineered DNA may reflect the low number of principal components visualised and hence, the variance explained by the first two principal components.

To further assess whether natural and engineered DNA can have distinct k-mer signatures, natural plasmid and engineered vector sequences were clustered into two groups using hierarchical clustering. Overall, increasing the value of k led to more homogeneous groups (Supplementary Table S1). Specifically, the clustering purity ranged from 0.62 (for 3-mer) and 0.86 (for 9-mer). Despite the high homogeneity of the groups with 9-mer signatures, clustering natural plasmid and engineered vector sequences was computationally demanding (Supplementary Figure S5). In comparison, 7-mer signatures offered a good trade-off between sequence specificity and computational resources. This implies that 7-mer signatures of natural and engineered DNA were sufficiently unique, and therefore chosen for further investigation.

Given that natural and engineered DNA can have distinct 7-mer signatures, we then investigated precisely which 7-mer sequences were different between natural and engineered DNA. To do this, we examined the loadings of every 7-mer sequence on the first two principal components, which measures the importance of each 7-mer sequence on a particular principal component. In general, we found relatively high loadings for both AT-rich (i.e., only contains A’s or T’s) and GC-rich (i.e., only contains C’s or G’s) 7-mer sequences on the first two principal components (Supplementary Figure S6). Further investigation revealed that engineered vector sequences contained significantly fewer AT-rich (two-sided *t*-test *p*-value <0.001) and significantly more GC-rich (two-sided *t*-test *p*-value <0.001) 7-mer sequences compared to natural plasmid sequences. Consistent with this was the higher GC content of engineered vector sequences (49.1%) compared to natural plasmid sequences (45.9%). Interestingly, across the different host species, we also noted that the average and median GC content of natural plasmid sequences ranged was 45.9% and 44.8%, respectively. This suggests that the lower GC content of natural plasmid sequences was not due to their host species. Instead, differences in GC content were likely due to the different genetic elements present within engineered vector and natural plasmid sequences, thereby resulting in k-mer signatures that are unique to natural and engineered DNA.

In addition to AT-rich and GC-rich 7-mer sequences, we found other 7-mer sequences with high loadings on the first two principal components (Supplementary Figure S6). Upon examining the 25 highest loaded 7-mer sequences that were not AT-rich or GC-rich, we found that the frequencies for most of these 7-mer sequences (22/25) were on average higher in engineered vector sequences compared to natural plasmid sequences. Interestingly, we noted that these 7-mer sequences were often found in regions that are crucial to engineered constructs but difficult to identify in nature due to their lack of sequence motifs, such as the origin of replication and promoter regions. This was expected since different engineered constructs are likely to contain the same well-defined sequence features, resulting in a frequency bias towards certain oligonucleotides of engineered vector sequences compared to natural plasmid sequences. Together, the above demonstrates that different sequence design elements can lead to distinct k-mer signatures and highlights the potential to distinguish between natural and engineered DNA based on their unique k-mer signatures.

### 3.2 Overview of Synsor

Having established that natural and engineered DNA could have distinct k-mer signatures and that their 7-mer signatures were sufficiently unique, we then developed Synsor, a tool for identifying engineered DNA sequences. To accomplish this, Synsor uses an artificial neural network to classify whether a DNA sequence is natural or engineered. By querying suspected sequences against the model, Synsor can identify sequences that are likely to have been engineered.

**FIGURE 2:**
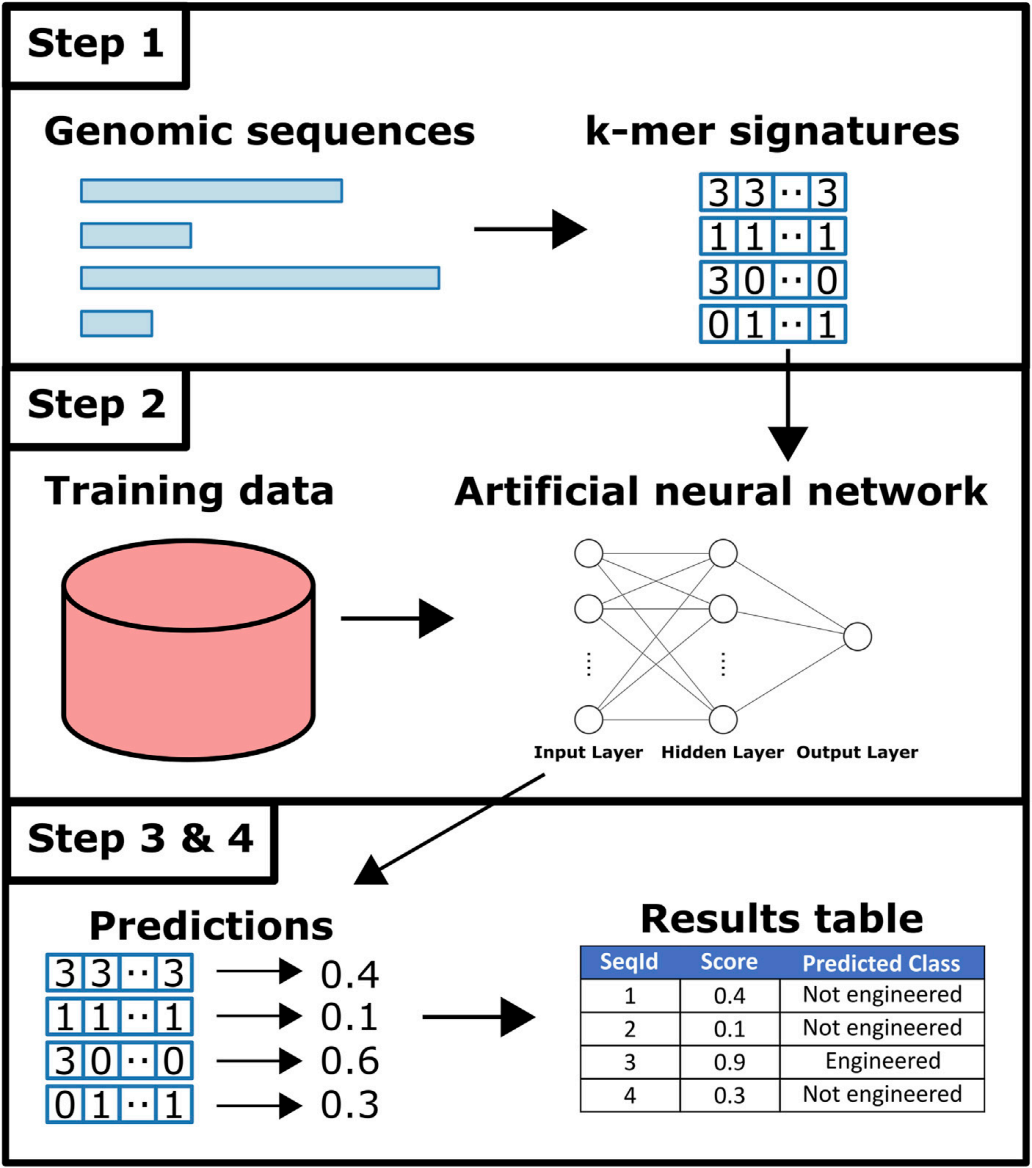
Overview of Synsor. Full description in Materials and Methods section. 1) Unique k-mer frequencies calculated for each sequence. 2) Sequences queried against an artificial neural network trained on 7-mer signatures of known natural plasmid and engineered vector sequences from NCBI. 3) and 4) Engineered DNA sequences are identified and tabularised.

An overview of Synsor is shown in Figure 2. Synsor requires a list of genomic sequences in FASTA format. Sequences can be fully sequenced genomes, or contigs from genome assembly. Analysing the input sequences with Synsor involves the following steps. 1) Variable length sequences in FASTA file are encoded into fixed-length 7-mer signatures. 2) 7-mer signatures of sequences are queried against an artificial neural network. For a full description of the predictive model, see the Materials and Methods section. 3) For each sequence, Synsor outputs a score between 0 and 1, and sequences with a score >0.5 were considered “engineered”. 4) The results of step 3 are recorded in the output tab-separated (TSV) file.

To evaluate the performance of Synsor, we analysed natural plasmid and engineered vector sequences that were never used to train the model (i.e., testing set) and in effect, were considered unknown. This was done by calculating the number of engineered vector sequences that were predicted to be “engineered” (true positives, TP), and “not engineered” (false negatives, FN), and the number of natural plasmid sequences that were predicted to be “engineered” (false positives, FP) and “not engineered” (true negatives, TN). The total of each metric was then used to calculate the accuracy, precision and recall of the model, where accuracy is defined as $\frac{\left(TP+TN\right)}{\left(TP+FP+TN+FN\right)}$ , precision is defined as $\frac{TP}{\left(TP+FP\right)}$ and recall is defined as $\frac{TP}{\left(TP+FN\right)}$ .

Almost all the engineered vector sequences (1,977/1,986) and almost all the natural plasmid sequences (1,698/1,709) in the testing set were correctly classified and hence, corresponds to true positives and true negatives, respectively. We also found 9 engineered vector sequences and 11 natural plasmid sequences in the testing set that were incorrectly classified and hence, corresponds to false negatives and false positives, respectively. From these, we calculated that the accuracy, precision and recall of Synsor was 0.994, 0.994, and 0.995, respectively. Overall, these results demonstrate the capacity of Synsor to accurately identify engineered DNA sequences based on their k-mer signatures.

We also investigated whether the high accuracy, precision and recall of Synsor could be explained by the highly similar sequences during training, or by the host species of the sequences. To do this, natural plasmid and engineered vector sequences were separately clustered into groups with MMseqs2, using different sequence identity thresholds (ranging between 0.2 and 0.8). For each sequence identity threshold, representative sequences from each group were randomly partitioned into an 80% training and 20% testing set. 7-mer signatures of sequences in the training and testing set were then used to train and evaluate the performance of Synsor, and the performance of different classifiers for species classification for each sequence identity threshold. Across the different sequence identity thresholds (Supplementary Figure S7), we found that the accuracy, precision, and recall of Synsor were consistently high (i.e., > 0.97). Meanwhile, every model for species classification failed to correctly predict the species of any sequences in the testing set. Together, the above suggests that the identification of engineered DNA sequences by Synsor was not due to the memorization of highly similar sequences during training or host species classification.

### 3.3 Case study 1: detecting potential genetically engineered organisms

Having established that engineered DNA could be identified by Synsor, we then investigated whether Synsor could be used to detect organisms that may have been genetically engineered. To do this, we analysed experimental genomic data for previously known genetically engineered yeast strains (Collins et al., 2021). Engineered vector constructs reported in the original study were known prior to genetic engineering. However, for this study, we assumed no prior knowledge about the genome of the host organism, the engineered vector construct or knowledge of genetic engineering. For comparison, we also analysed experimental genomic data for previously known natural yeast strains (Giordano et al., 2017). For each genomic dataset, paired-end reads were *de novo* assembled into contigs with SPAdes using default parameters. To ensure that predictions by Synsor were accurate and reliable, contigs shorter than 2.5 kb were removed from each *de novo* assembled genome, and the resulting contigs were analysed with Synsor.

**FIGURE 3:**
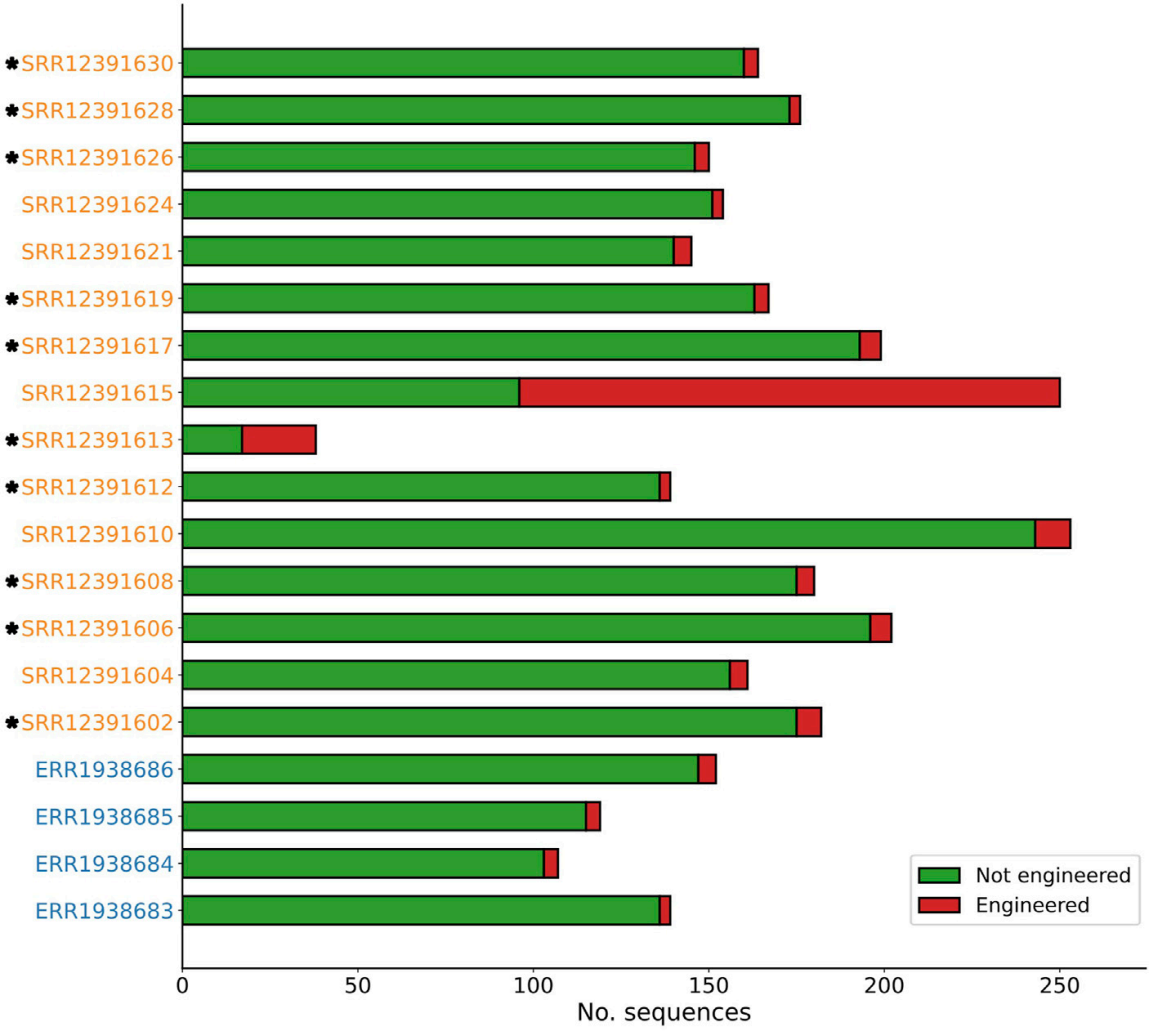
Stacked bar plot showing the distribution of contigs predicted to be engineered or not with Synsor for genomic datasets of known natural (blue) and genetically engineered (orange) yeast strains. Each segment in the column represents the number of contigs predicted to be engineered (red) or not engineered (green). NCBI BLAST results for contigs predicted to be engineered revealed at least one contig associated with an engineered vector construct in 10 out of 15 genomic datasets (indicated by *).

Across four genomic datasets of natural yeast strains, a total of 517 contigs were analysed by Synsor, with the length of these contigs ranging from 2,531 to 3,39,928 (Figure 3). Of the 517 contigs, Synsor classified 16 (3.1%) contigs as “engineered”, with the number of contigs in each of the four datasets ranging from 3 (2.1%) and 5 (3.2%). The average and median number of contigs classified as “engineered” was 4 and 4, respectively. By contrast, across fifteen genomic datasets of genetically engineered yeast strains, a total of 2,560 contigs were analysed by Synsor, with the length of contigs ranging from 2,514 to 1,363,605 bases. Of the 2,560 contigs, Synsor classified 240 (9.3%) contigs as “engineered”, with the number of contigs in each of the fifteen datasets ranging from 3 (1.7%) and 154 (61.6%). The average and median number of contigs classified as “engineered” was 16 and 5, respectively. A list of the number of contigs classified as “engineered” for each genomic dataset is included in Supplementary Table S2.

We then verified whether the contigs identified by Synsor were in fact engineered. To do this, contigs that were classified as “engineered” were aligned to the non-redundant nucleotide database using NCBI BLAST. Across the four genomic datasets of natural yeast strains, we found that all 16 contigs were not associated with engineered vector constructs and instead were associated with natural chromosomal DNA from yeast (Supplementary Table S3). By contrast, across the fifteen genomic datasets of genetically engineered yeast strains, we found 11 contigs that were associated with engineered vector constructs. Notably, NCBI BLAST revealed that these contigs were the same as those described in the original paper, confirming that Synsor can identify engineered DNA sequences. More interesting was the observation that contigs associated with engineered vector constructs were not exact copies of sequences in the training set, highlighting a capacity to identify engineered DNA sequences that do not exactly match known sequences with Synsor. In addition to contigs associated with engineered vector constructs, we found 229 contigs that were associated with natural chromosomal DNA from yeast. However, most of these contigs (184/229) were from samples SRR12391610, SRR12391613 and SRR12391615, suggesting that the natural DNA of some genomes will make it difficult to detect engineered DNA. Nonetheless, our results demonstrate that Synsor can reduce the search space to a few candidate sequences that may have been engineered, thereby streamlining the process of identifying engineered DNA sequences in whole genome sequencing data.

To further evaluate the performance of Synsor, the number of contigs associated with engineered vector constructs across the fifteen genomic datasets of genetically engineered yeast strains were compared against those from INSIDER (Tay et al., 2021), a tool for detecting foreign DNA sequences. Of the 2,560 contigs classified as “engineered”, we found 7 contigs with INSIDER that were associated with engineered vector constructs. The lower number of contigs identified by INSIDER compared to Synsor (11) suggests that INSIDER was less effective at identifying engineered DNA, highlighting the importance of a more targeted approach.

We finally investigated whether Synsor could help in assessing the extent of genetic engineering present within a genome. Of the 15 genomic datasets of genetically engineered yeast strains, we found that 10 (66.6%) datasets contained at least one contig associated with an engineered vector construct, with the number of contigs in each of the 10 datasets ranging from 1 to 2 (Figure 3). The low number of contigs associated with engineered vector constructs found likely reflects the fact that only a single vector sequence was transformed into each yeast strain. For the remaining 5 datasets that did not contain contigs associated with engineered vector constructs, further investigation revealed several contigs were in fact associated with engineered vector constructs but were either incorrectly classified by Synsor or were removed due to their relatively short length. This highlights that some engineered DNA will be missed due to the technical limitations of short read sequencing. Nevertheless, these results demonstrate that Synsor can help to assess the extent of genetic engineering present within a genome and determine whether the genome of an organism has been artificially manipulated. Importantly, Synsor required no prior knowledge about the genome, the engineered DNA sequence or knowledge of genetic engineering, thereby allowing for enhanced detection of potential biothreats.

### 3.4 Case study 2: locating where engineered DNA is being introduced into the environment

Accidental or deliberate introduction of engineered DNA into the environment poses a risk that could endanger human health, disrupt agricultural production, or cause lasting ecosystem harm. Monitoring sewage for engineered DNA in liquid waste can help in determining whether engineered DNA is present in the environment and thus, the potential geographical source of genetic engineering. Here, we investigated whether Synsor could be used to locate where engineered DNA is being introduced into the environment.

To demonstrate how Synsor can be used to locate where engineered DNA is being introduced into the environment, we simulated the introduction of a genetically engineered organism to the environment (referred to as ST-V). This was done by combining a metagenomic sample taken from a wastewater treatment plant (Che et al., 2019), with a genomic dataset for a previously known genetically engineered *E. coli* strain (Ames et al., 2019). For comparison, we also obtained metagenomic samples taken from two other wastewater treatment plants (referred to as SWH and STL). In effect, we analysed metagenomic samples taken from wastewater treatment plants in three different geographical locations. For each metagenomic dataset, paired-end reads were *de novo* assembled into contigs with metaSPAdes using default parameters. After removing contigs shorter than 2.5 kb from each *de novo* assembled metagenome, the resulting contigs were analysed with Synsor.

**FIGURE 4:**
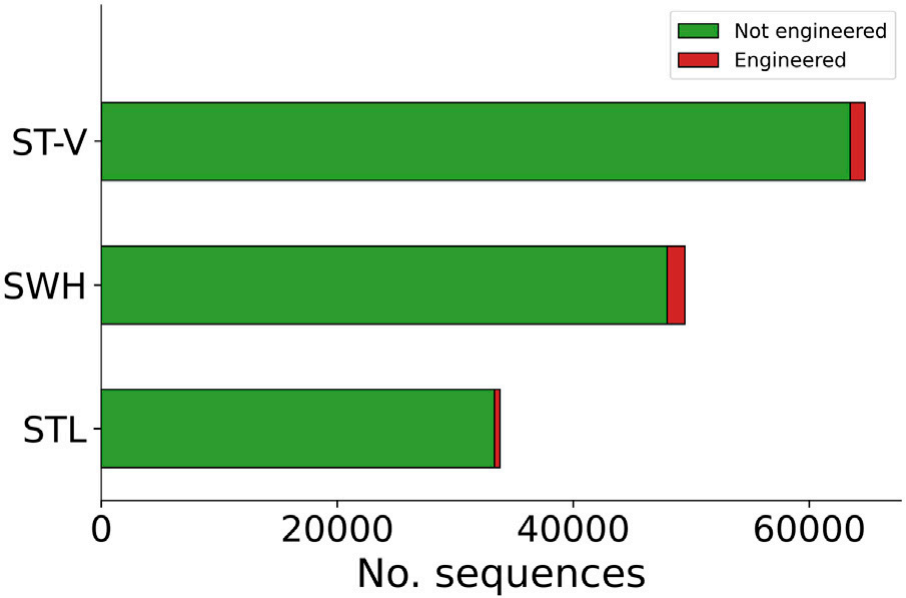
Stacked bar plot showing the distribution of contigs predicted to be engineered or not with Synsor for each metagenomic sample. Each segment in the column represents the number of contigs predicted to be engineered (red) or not engineered (green). NCBI BLAST results for contigs predicted to be engineered revealed 1 contig associated with an engineered vector construct in sample ST-V, but no contigs in samples SWH or STL.

For samples SWH and STL, a total of 49,480 contigs and 33,844 contigs were analysed with Synsor, respectively (Figure 4). The length of these contigs ranged from 2,501 bases and 5,53,601 bases. For sample SWH, Synsor classified 1,503 (3.0%) contigs as “engineered”. On the other hand, for sample STL, Synsor classified 466 (1.6%) contigs as “engineered”. By contrast, for sample ST-V, a total of 64,705 contigs were analysed with Synsor, with the length of these contigs ranging from 2,501 bases to 491,013 bases. Of the 64,705 contigs, Synsor classified 1,250 (1.9%) contigs as “engineered”. A list of the number of contigs classified as “engineered” for each metagenomic sample is included in Supplementary Table S4.

Having prioritized the candidates of interest, we then verified whether the contigs identified by Synsor were in fact engineered. To do this, we aligned contigs that were classified as “engineered” to the non-redundant nucleotide database using NCBI BLAST. For samples SWH and STL, we found that all 1,503 and 466 contigs were not associated with engineered vector constructs, respectively. Instead, most of the contigs from sample SWH (1,311/1,503) and (355/466) STL were associated with natural DNA from bacterial and viral species typically found in wastewater, with the remainder returning no hits. By contrast, for sample ST-V, we found 1 contig associated with the same engineered vector construct described in the original study (Supplementary Table S5), confirming that Synsor can detect the presence of engineered DNA in the environment. Note, however, that this likely reflects the fact that only a single vector sequence was transformed into the *E. coli* strain and subsequently introduced into the wastewater sample. In addition, we found 1,002 contigs that were associated with natural DNA from bacterial and viral species typically found in wastewater, and 247 contigs that returned no hits with NCBI BLAST. Nonetheless, the above results illustrate that Synsor can serve as a fast prefilter step and help in determining whether engineered DNA is present in the environment, highlighting the potential of using Synsor to monitor engineered biothreats in environmental systems.

We finally investigated whether Synsor could help in locating where engineered DNA is being introduced into the environment. Of the 3 metagenomic samples, we found that 1 sample contained an engineered vector construct, namely, ST-V. The identification of engineered DNA in sample ST-V but not in samples SWH or STL, suggests that engineered DNA was introduced nearby one wastewater treatment plant (i.e., Shatin) but not the others (i.e., Shek Wu Hui or Stanley). Overall, these results illustrate that Synsor can help to reduce the search space to a few candidate locations, and thus highlight the potential of using this approach to locate where engineered DNA is being introduced into the environment.

## 4 Discussion

In this study, we presented Synsor, a tool for identifying engineered DNA sequences in high-throughput sequencing data. Synsor leverages k-mer signature differences between naturally occurring and engineered DNA sequences and uses an artificial neural network to classify whether a DNA sequence is natural or engineered. By querying suspected sequences against the model, Synsor can identify sequences that are likely to have been engineered. As a proof-of-concept, we demonstrated how Synsor can be used to detect potential genetically engineered organisms and locate where engineered DNA is being introduced into the environment. Critically, in both case studies, Synsor required no prior knowledge about the genome or metagenomic sample, the engineered DNA sequence or knowledge of genetic engineering. For this reason, Synsor could be readily used to identify engineered DNA in completely novel biological or environmental systems.

Synsor is one of the first tools specifically designed for identifying engineered DNA sequences that does not rely on sequence alignment. In doing so, this approach has the potential to identify engineered DNA sequences that are not publicly available, especially those engineered for bioterrorism. Complementing existing alignment-based methods with alignment-free approaches will be useful for preventing the dissemination of synthetic sequences that could do harm but are missing from databases of known threats (Hoffmann et al., 2023). However, while Synsor is open-source and details of the tool are reported here, we note that it is possible to engineer specific sequences that will evade detection. In the future, a more comprehensive and closed-source version of Synsor should also be developed to help ensure that sequences cannot be engineered to bypass Synsor.

Predicting the engineering status of unknown DNA sequences with Synsor is dependent on the underlying sequences used to train the deep learning model. Without high quality DNA sequences, generalizing the model to predict the engineering status of novel sequences will be challenging. While not perfect, natural plasmid and engineered vector sequences remain useful representatives of natural and engineered DNA. However, given that not all natural DNA are plasmids, including natural chromosomal and viral sequences into the training set should help the model to identify natural DNA (Allen et al., 2008). The model could also benefit from engineered vector sequences curated in different online repositories such as AddGene (Kamens, 2015). Compared to NCBI, these repositories are likely to contain more engineered vector sequences and are thus more comprehensive. Despite this, access to the data must be requested and can be at the discretion of the curator, making it difficult to obtain engineered vector sequences and the associated metadata from these repositories. Nevertheless, including engineered vector sequences curated in different online repositories into the training set should help the model to identify engineered DNA (Nielsen and Voigt, 2018; Alley et al., 2020; Soares et al., 2022). Pruning sequences to only the most relevant elements to genetic engineering could also be useful for reducing the amount of noise in the training data, thereby improving the capacity of the model to detect differences between natural and engineered DNA that are associated with genetic engineering (Wang et al., 2021).

We showed that natural and engineered DNA can have distinct k-mer signatures. Despite this, classifying whether a DNA sequence is natural or engineered based on their k-mer signatures can be difficult. Longer k-mers will be of particular use, helping to distinguish between natural and engineered DNA sequences with highly similar k-mer signatures (Allen et al., 2008). Analysing longer k-mers could also be important for detecting the boundaries separating DNA from completely different species and help facilitate the identification of engineered chimeric sequences. However, it must be kept in mind that improvements to the performance of Synsor must be balanced with associated computational costs, as increasing the k-mer length will increase the number of variables and parameters involved. Reducing the large number of variables into a smaller dimensional space will be useful for identifying k-mers that can best discriminate between natural and engineered sequences and hence, better predict the engineering status of unknown DNA sequences (Meng et al., 2016). In the same way, combining different methods for identifying engineered DNA sequences will be useful for classifying sequences that are missed by any single approach (Crook et al., 2022; Adler et al., 2024; Berezin et al., 2024).

Although not investigated in this study, we anticipate that Synsor will be useful for identifying engineered DNA in data produced by long read sequencing technologies (Fraiture et al., 2018). The advantage of long read sequencing is that long contiguous regions of the genome can be sequenced in a single read, allowing engineered sequences to be recovered in their entirety (Amarasinghe et al., 2020). At the same time, compared to short reads, long reads can produce stable k-mer signatures (Sims et al., 2009). Querying long reads directly against Synsor should therefore lead to the identification of engineered reads and thus the detection of emerging biothreats without the need for genome assembly. However, compared to short reads, long reads can contain more errors which could affect the reliability of their k-mer signatures (Fraiture et al., 2018). Correcting errors with short reads will be useful for improving the quality of long read sequences, and thus the identification of engineered DNA with Synsor (Berbers et al., 2020).

As proof of concept, we demonstrated how Synsor can be used to monitor engineered DNA in wastewater, and how this could help in locating where these sequences are being introduced. Besides this, however, we envision that Synsor will also be useful for monitoring engineered DNA in a variety of environments, including airports and ports (Buytaers et al., 2021; D’aes et al., 2022). This could involve real-time sequencing of metagenomic samples on portable sequencing technologies such as the Nanopore MinION, and querying these metagenomic reads against Synsor. Performing separate analyses on reads that originated from known and unknown genomes could also help in prioritizing sequences for further analysis. Identifying engineered reads in this way should lead to the detection of engineered DNA in real-time, and therefore rapid detection of emerging biothreats. Meanwhile, quantifying the amount of engineered DNA introduced into these environments should also help in determining the extent of genetic engineering. An important application of this could be in measuring the effectiveness of biocontainment strategies, especially those implemented by institutional laboratories. However, it must be kept in mind that obtaining deep sequencing data of metagenomic samples from these complex environments in real-time remains an ongoing challenging (Latorre-Pérez et al., 2021). Adapting sequencing protocols towards specific environments will be necessary for improving the coverage of microbial communities, and thus the detection of engineered DNA (Latorre-Pérez et al., 2021).

In conclusion, we have developed Synsor, a tool for identifying engineered DNA sequences in high-throughput sequencing data. Through case studies from yeast and wastewater samples, we demonstrated how Synsor can be used to detect potential genetically engineered organisms and locate where engineered DNA sequences are being introduced into the environment. Synsor is therefore a powerful tool that will streamline the process of identifying engineered DNA in poorly characterized biological or environmental systems, thereby allowing for enhanced monitoring of emerging biothreats.

## Data Availability

The original contributions presented in the study are included in the article/Supplementary Material, further inquiries can be directed to the corresponding author.

## References

[B1] Adler A. Bader J. S. Basnight B. Booth B. W. Cai J. Cho E. (2024). Ensemble detection of DNA engineering signatures. ACS Synth. Biol. 13, 1105–1115. 10.1021/acssynbio.3c00398 38468602

[B2] Allen J. E. Gardner S. N. Slezak T. R. (2008). DNA signatures for detecting genetic engineering in bacteria. Genome Biol. 9, R56–R56.10. 10.1186/gb-2008-9-3-r56 18348716 PMC2397508

[B3] Alley E. C. Turpin M. Liu A. B. Kulp-McDowall T. Swett J. Edison R. (2020). A machine learning toolkit for genetic engineering attribution to facilitate biosecurity. Nat. Commun. 11, 6293–6312. 10.1038/s41467-020-19612-0 33293535 PMC7722865

[B4] Amarasinghe S. L. Su S. Dong X. Zappia L. Ritchie M. E. Gouil Q. (2020). Opportunities and challenges in long-read sequencing data analysis. Genome Biol. 21, 30. 10.1186/s13059-020-1935-5 32033565 PMC7006217

[B5] Ames J. R. Muthuramalingam M. Murphy T. Najar F. Z. Bourne C. R. (2019). Expression of different ParE toxins results in conserved phenotypes with distinguishable classes of toxicity. Microbiologyopen 8, e902. 10.1002/mbo3.902 31309747 PMC6813445

[B6] Berbers B. Saltykova A. Garcia-Graells C. Philipp P. Arella F. Marchal K. (2020). Combining short and long read sequencing to characterize antimicrobial resistance genes on plasmids applied to an unauthorized genetically modified Bacillus. Sci. Rep. 10, 4310. 10.1038/s41598-020-61158-0 32152350 PMC7062872

[B7] Berezin C. T. Peccoud S. Kar D. M. Peccoud J. (2024). Cryptographic approaches to authenticating synthetic DNA sequences. Trends Biotechnol. S0167-7799, 00031–3. 10.1016/j.tibtech.2024.02.002 PMC11309913 38418329

[B8] Buytaers F. E. Fraiture M.-A. Berbers B. Vandermassen E. Hoffman S. Papazova N. (2021). A shotgun metagenomics approach to detect and characterize unauthorized genetically modified microorganisms in microbial fermentation products. Food Chem. Mol. Sci. 2, 100023. 10.1016/j.fochms.2021.100023 PMC8991599 35415629

[B9] Che Y. Xia Y. Liu L. Li A.-D. Yang Y. Zhang T. (2019). Mobile antibiotic resistome in wastewater treatment plants revealed by Nanopore metagenomic sequencing. Microbiome 7, 44. 10.1186/s40168-019-0663-0 30898140 PMC6429696

[B10] Collins J. H. Keating K. W. Jones T. R. Balaji S. Marsan C. B. Çomo M. (2021). Engineered yeast genomes accurately assembled from pure and mixed samples. Nat. Commun. 12, 1485–1515. 10.1038/s41467-021-21656-9 33674578 PMC7935868

[B11] Crook O. M. Warmbrod K. L. Lipstein G. Chung C. Bakerlee C. W. McKelvey T. G. (2022). Analysis of the first genetic engineering attribution challenge. Nat. Commun. 13, 7374. 10.1038/s41467-022-35032-8 36450726 PMC9712580

[B12] D’aes J. Fraiture M.-A. Bogaerts B. De Keersmaecker S. C. J. Roosens N. H. C. J. Vanneste K. (2022). Metagenomic characterization of multiple genetically modified Bacillus contaminations in commercial microbial fermentation products. Life 12, 1971. 10.3390/life12121971 36556336 PMC9781105

[B13] Fraiture M.-A. Herman P. Taverniers I. Loose M. D. Deforce D. Roosens N. H. (2015). Current and new approaches in GMO detection: challenges and solutions. Biomed. Res. Int. 2015, 1–22. 10.1155/2015/392872 PMC4624882 26550567

[B14] Fraiture M.-A. Saltykova A. Hoffman S. Winand R. Deforce D. Vanneste K. (2018). Nanopore sequencing technology: a new route for the fast detection of unauthorized GMO. Sci. Rep. 8, 7903. 10.1038/s41598-018-26259-x 29785005 PMC5962636

[B15] Gargis A. S. Cherney B. Conley A. B. McLaughlin H. P. Sue D. (2019). Rapid detection of genetic engineering, structural variation, and antimicrobial resistance markers in bacterial biothreat pathogens by Nanopore sequencing. Sci. Rep. 9, 13501–13514. 10.1038/s41598-019-49700-1 31534162 PMC6751186

[B16] Giordano F. Aigrain L. Quail M. A. Coupland P. Bonfield J. K. Davies R. M. (2017). *De novo* yeast genome assemblies from MinION, PacBio and MiSeq platforms. Sci. Rep. 7, 3935–4010. 10.1038/s41598-017-03996-z 28638050 PMC5479803

[B17] Hoffmann S. A. Diggans J. Densmore D. Dai J. Knight T. Leproust E. (2023). Safety by design: biosafety and biosecurity in the age of synthetic genomics. iScience 26, 106165. 10.1016/j.isci.2023.106165 36895643 PMC9988571

[B18] Hughes R. A. Ellington A. D. (2017). Synthetic DNA synthesis and assembly: putting the synthetic in synthetic biology. Cold Spring Harb. Perspect. Biol. 9, a023812. 10.1101/cshperspect.a023812 28049645 PMC5204324

[B19] Kamens J. (2015). The Addgene repository: an international nonprofit plasmid and data resource. Nucleic Acids Res. 43, D1152–D1157. 10.1093/nar/gku893 25392412 PMC4384007

[B20] Karlin S. Mrázek J. Campbell A. M. (1997). Compositional biases of bacterial genomes and evolutionary implications. J. Bacteriol. 179, 3899–3913. 10.1128/jb.179.12.3899-3913.1997 9190805 PMC179198

[B21] Latorre-Pérez A. Pascual J. Porcar M. Vilanova C. (2021). A lab in the field: applications of real-time, *in situ* metagenomic sequencing. Biol. Methods Protoc. 5, bpaa016. 10.1093/biomethods/bpaa016 PMC7585387 33134552

[B22] Meng C. Zeleznik O. A. Thallinger G. G. Kuster B. Gholami A. M. Culhane A. C. (2016). Dimension reduction techniques for the integrative analysis of multi-omics data. Brief. Bioinform. 17, 628–641. 10.1093/bib/bbv108 26969681 PMC4945831

[B23] Nielsen A. A. K. Voigt C. A. (2018). Deep learning to predict the lab-of-origin of engineered DNA. Nat. Commun. 9, 3135–3210. 10.1038/s41467-018-05378-z 30087331 PMC6081423

[B24] Ruttink T. Demeyer R. Van Gulck E. Van Droogenbroeck B. Querci M. Taverniers I. (2010). Molecular toolbox for the identification of unknown genetically modified organisms. Anal. Bioanal. Chem. 396, 2073–2089. 10.1007/s00216-009-3287-6 19937431

[B25] Sims G. E. Jun S. R. Wu G. A. Kim S. H. (2009). Alignment-free genome comparison with feature frequency profiles (FFP) and optimal resolutions. Proc. Natl. Acad. Sci. U. S. A. 106, 2677–2682. 10.1073/pnas.0813249106 19188606 PMC2634796

[B26] Soares I. M. Camargo F. H. F. Marques A. Crook O. M. (2022). Improving lab-of-origin prediction of genetically engineered plasmids via deep metric learning. Nat. Comput. Sci. 2, 253–264. 10.1038/s43588-022-00234-z 38177551

[B27] Tay A. P. Hosking B. Hosking C. Bauer D. C. Wilson L. O. W. (2021). INSIDER: alignment-free detection of foreign DNA sequences. Comput. Struct. Biotechnol. J. 19, 3810–3816. 10.1016/j.csbj.2021.06.045 34285780 PMC8273350

[B28] Wang F. Zhang W. (2019). Synthetic biology: recent progress, biosafety and biosecurity concerns, and possible solutions. J. Biosaf. Biosecur. 1, 22–30. 10.1016/j.jobb.2018.12.003

[B29] Wang Q. Kille B. Liu T. R. Elworth R. A. L. Treangen T. J. (2021). PlasmidHawk improves lab of origin prediction of engineered plasmids using sequence alignment. Nat. Commun. 12, 1167–1212. 10.1038/s41467-021-21180-w 33637701 PMC7910462

[B30] Zielezinski A. Vinga S. Almeida J. Karlowski W. M. (2017). Alignment-free sequence comparison: benefits, applications, and tools. Genome Biol. 18, 186–217. 10.1186/s13059-017-1319-7 28974235 PMC5627421

