# Supplementary material for: Synsor: a tool for alignment-free detection of engineered DNA sequences
Source: Front Bioeng Biotechnol. 2024 Jul 12;12:1375626. doi: 10.3389/fbioe.2024.1375626 (PMC11272466; doi:10.3389/fbioe.2024.1375626)
Supplement: Supplementary file 2 [file DataSheet1.docx]

Supplementary Material


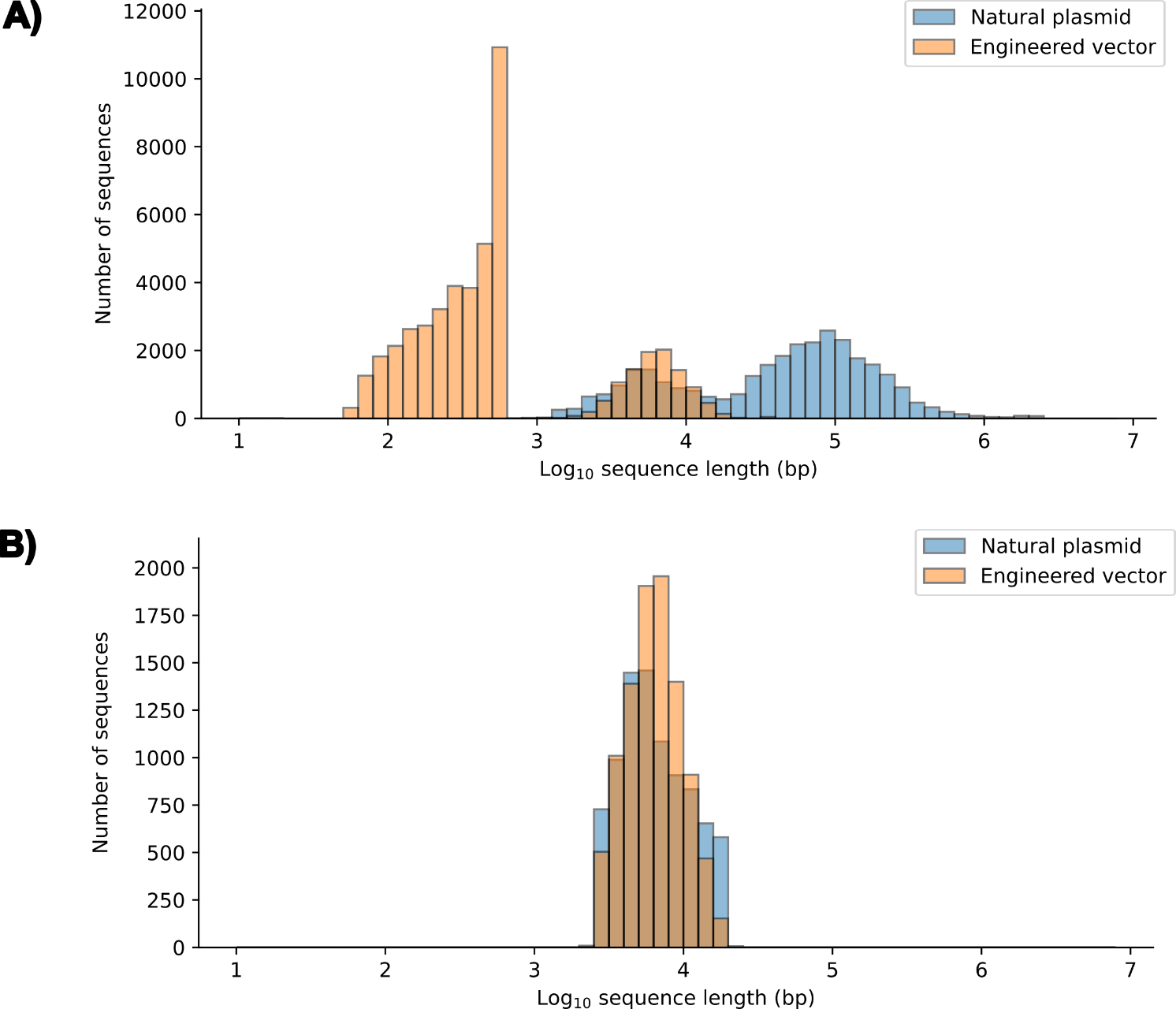


**Figure S1:** Histogram of sequence lengths for natural plasmid (blue) and engineered vector (orange) sequences (A) before and (B) after filtering.


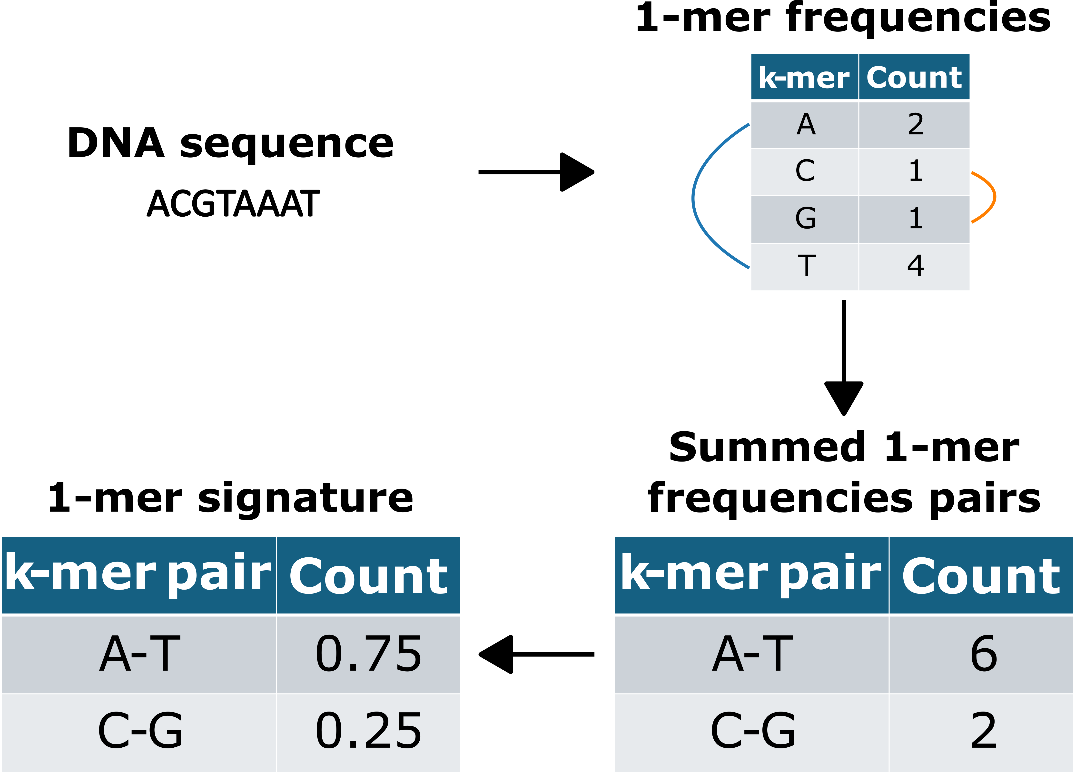


**Figure S2**: Overview of workflow used to calculate k-mer signatures. It involves identifying all possible subsequences of a given length (i.e., k-mer) and counting the frequency of each k-mer. The frequency of each k-mer and its reverse complement (indicated by blue and orange lines) were then summed. The relative proportion for each k-mer pair was then calculated, resulting in a relative frequency vector.


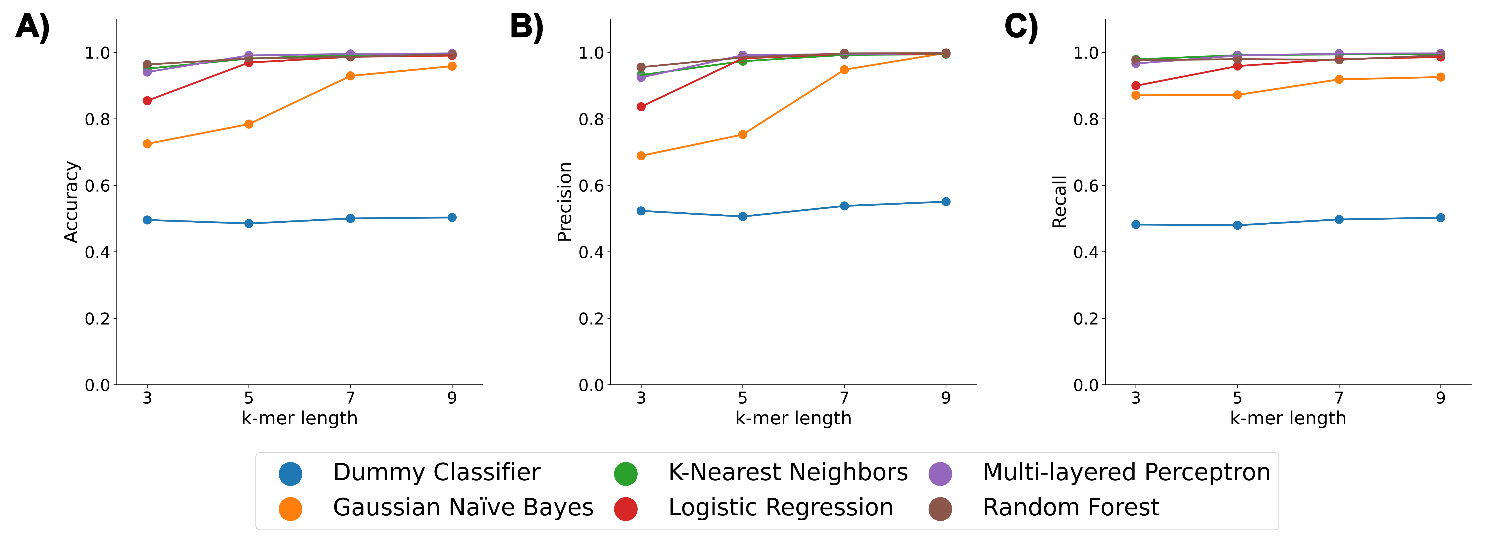


**Figure S3:** Line plots comparing the A) accuracy, B) precision and C) recall obtained by different classification algorithms for different values of k. The colour of each line corresponds to a specific classification algorithm including Dummy Classifier which makes predictions at random (blue), Gaussian Naïve Bayes (orange), K-Nearest Neighbors (green), Logistic Regression (red), Multi-layered Perceptron (purple) and Random Forest (brown). Overall, increasing the value of k increased the accuracy, precision and recall obtained by each classifier, and thus their performance. However, the accuracy, precision and recall of each classifier levelled off with increases in the value of k.


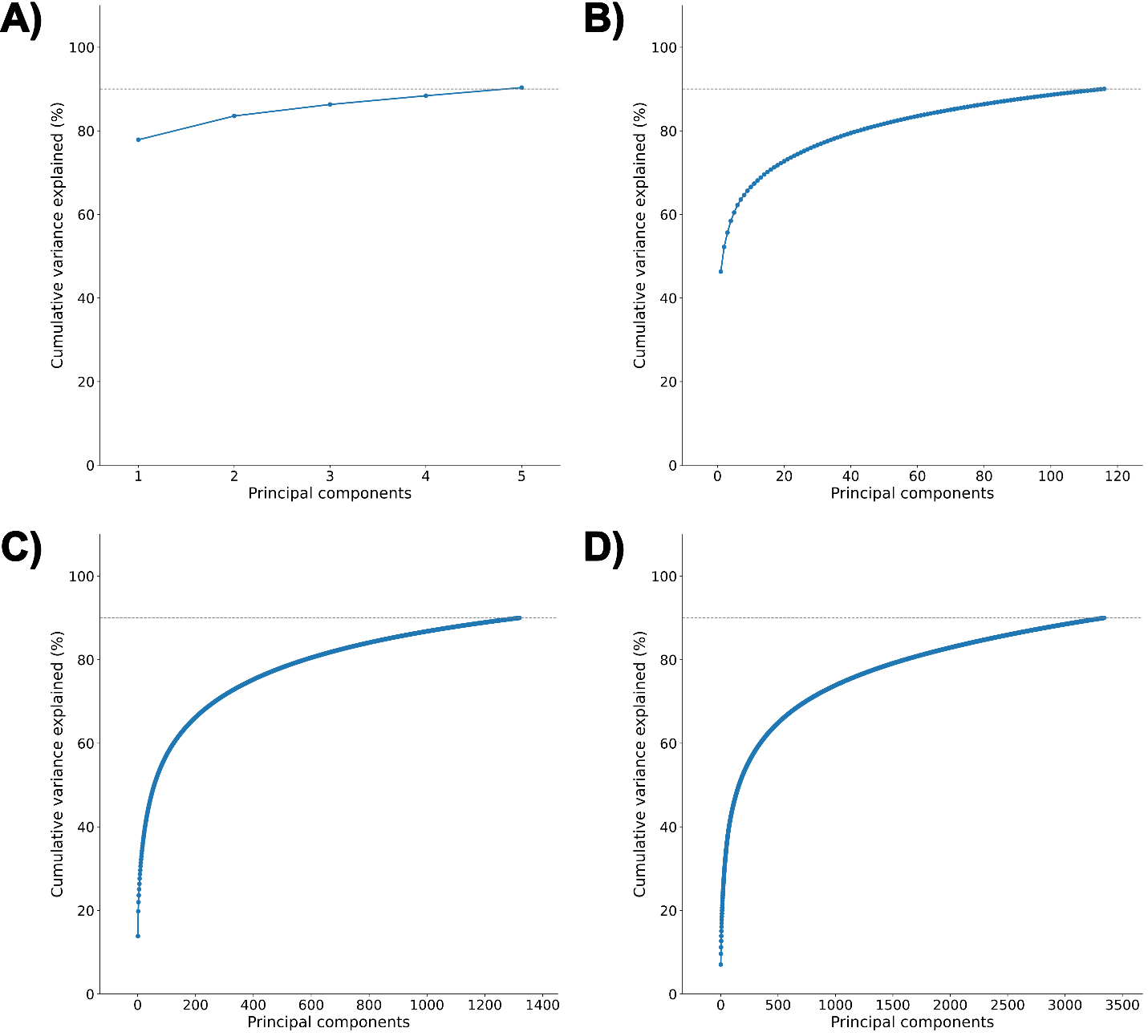


**Figure S4**: Line plots showing the cumulative variance explained by the principal components resulting from PCA on the A) 3-mer, B) 5-mer, C) 7-mer and D) 9-mer signatures of natural plasmid and engineered vector sequences for different values of k. Overall, increasing the dimensionality of the k-mer vector space increases the number of principal components required to represent at least 90% (grey line) of the total variance.


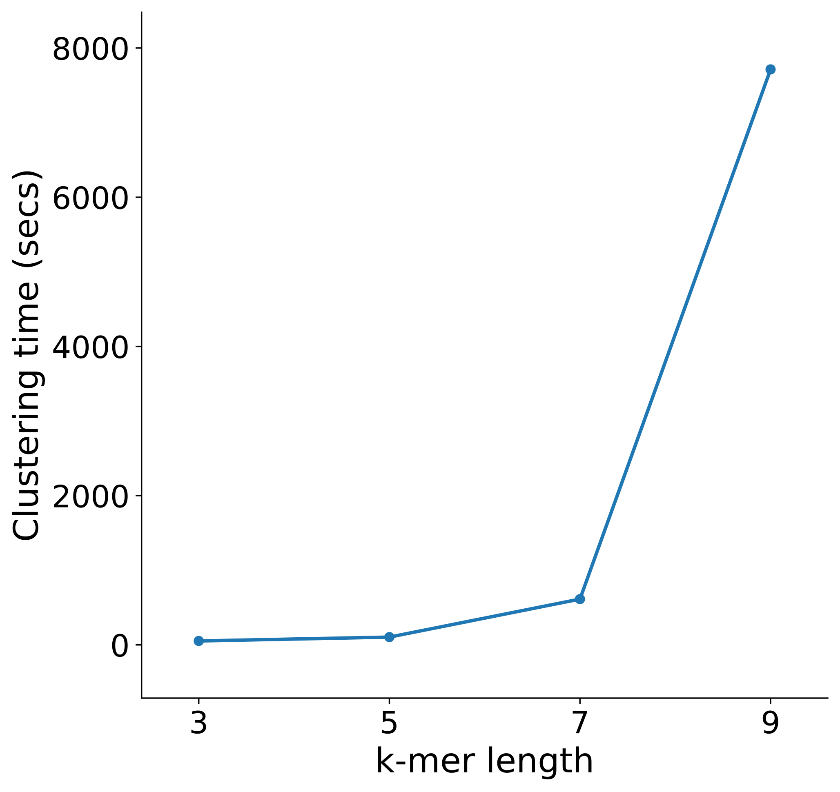


**Figure S5:** Line plots showing the time spent clustering natural plasmid and engineered vector sequences into two groups for different values of k. Overall, increasing the value of k increased the time spent on clustering sequences.


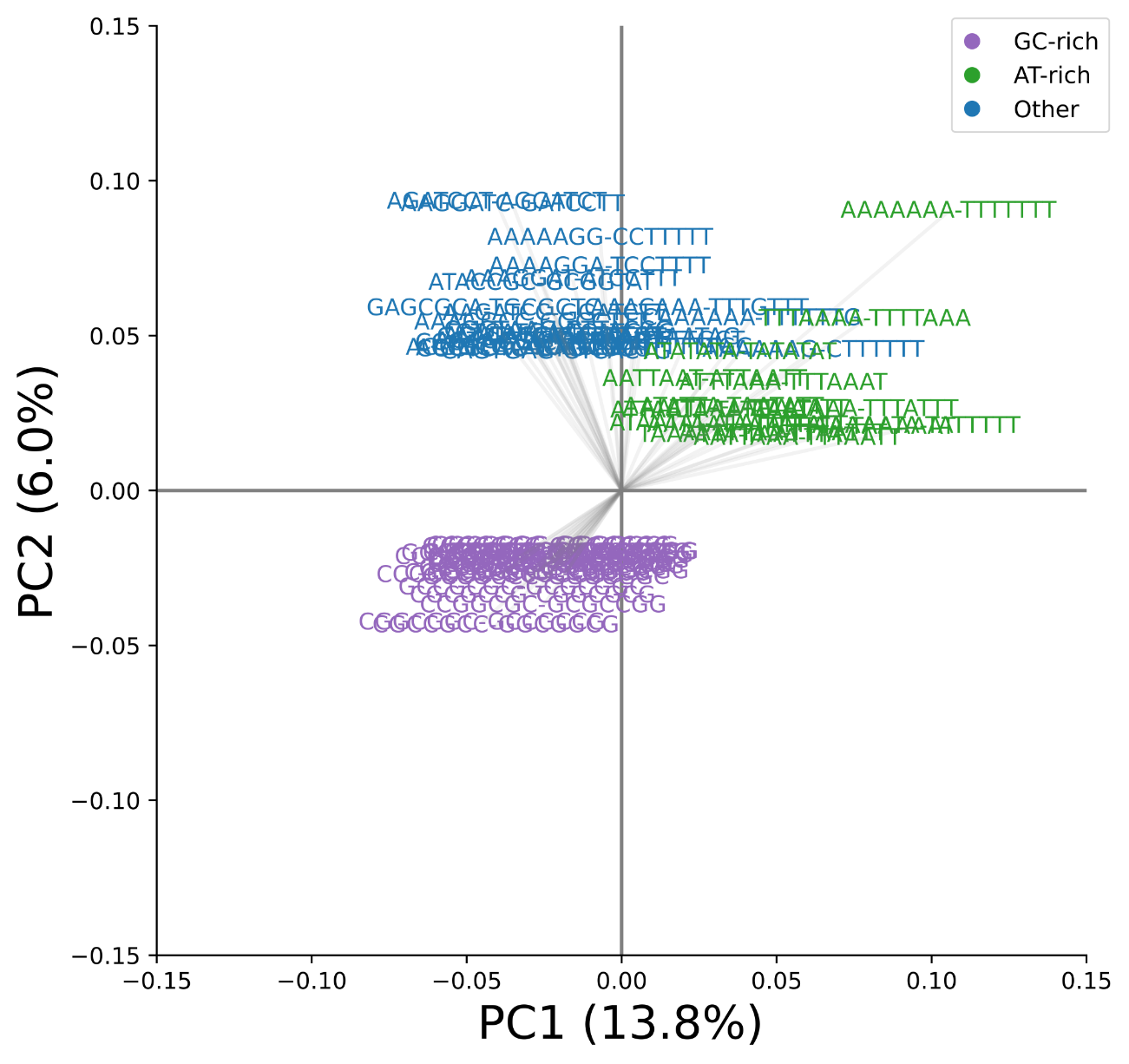


**Figure S6**: Loadings on the first two principal components resulting from the PCA on 7-mer signatures of natural plasmid and synthetic vector sequences. Loadings for AT-rich (green) and GC-rich (purple) 7-mer sequences, and for the 25 highest loaded 7-mer sequences that were not AT-rich or GC-rich (blue). Loadings for all other 7-mer sequences were removed to facilitate visualisation.


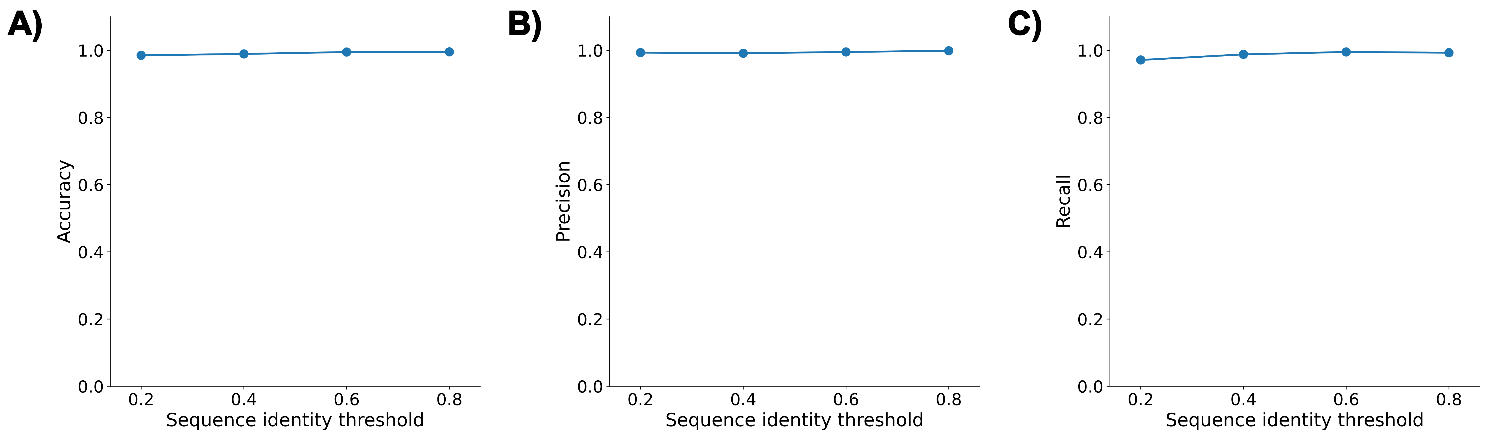


**Figure S7**: Line plots comparing the A) accuracy, B) precision and C) recall obtained by Synsor using representative natural plasmid and engineered vector sequences at different sequence identity thresholds. Overall, the accuracy, precision and recall of Synsor was consistently high across the different sequence identity thresholds, suggesting that Synsor can correctly predict the engineering status of sequences even after extremely similar sequences were removed.
